# Anti-Allergic Effects of *Myrciaria dubia* (Camu-Camu) Fruit Extract by Inhibiting Histamine H1 and H4 Receptors and Histidine Decarboxylase in RBL-2H3 Cells

**DOI:** 10.3390/antiox11010104

**Published:** 2021-12-31

**Authors:** Nhung Quynh Do, Shengdao Zheng, Sarang Oh, Quynh T. N. Nguyen, Minzhe Fang, Minseon Kim, Junhui Choi, Myeong-Ju Kim, Jeehaeng Jeong, Tae-Hoo Yi

**Affiliations:** 1 Graduate School of Biotechnology, Kyung Hee University, 1732 Deogyeong-daero, Giheung-gu, Yongin-si 17104, Korea; quynhnhung96@khu.ac.kr (N.Q.D.); sdjeong0719@khu.ac.kr (S.Z.); quynhnguyen@khu.ac.kr (Q.T.N.N.); mincheol1030@khu.ac.kr (M.F.); dbs03067@khu.ac.kr (M.K.); junhi4703@khu.ac.kr (J.C.); espritmj@khu.ac.kr (M.-J.K.); 2 Snow White Factory Co., Ltd., 807 Nonhyeonro, Gangnam-gu, Seoul 06032, Korea; blazma@kmu.ac.kr (S.O.); dbensk0205@khu.ac.kr (J.J.)

**Keywords:** camu-camu fruit extract, anti-allergic effect, histamine H1 receptor, histamine H4 receptor, histidine decarboxylase, RBL-2H3 cells

## Abstract

Although *Myrciaria dubia* (camu-camu) has been shown to exert anti-oxidant and anti-inflammatory effects in both in vitro and in vivo studies, its use in allergic responses has not been elucidated. In the present study, the anti-allergic effect of 70% ethanol camu-camu fruit extract was tested on calcium ionophore (A23187)-induced allergies in RBL-2H3 cells. The RBL-2H3 cells were induced with 100 nM A23187 for 6 h, followed by a 1 h camu-camu fruit extract treatment. A23187 sanitization exacerbated mast cell degranulation; however, camu-camu fruit extract decreased the release of histamine and β-hexosaminidase, which are considered as key biomarkers in cell degranulation. Camu-camu fruit extract inhibited cell exocytosis by regulating the calcium/nuclear factor of activated T cell (NFAT) signaling. By downregulating the activation of mitogen-activated protein kinase (MAPK) signaling, camu-camu fruit extract hindered the activation of both histamine H1 and H4 receptors and inhibited histidine decarboxylase (HDC) expression by mediating its transcription factors KLF4/SP1 and GATA2/MITF. In A23187-induced ROS overproduction, camu-camu fruit extract activated nuclear factor erythroid-2-related factor 2 (Nrf2) to protect mast cells against A23187-induced oxidative stress. These findings indicate that camu-camu fruit extract can be developed to act as a mast cell stabilizer and an anti-histamine. This work also “opens the door” to new investigations using natural products to achieve breakthroughs in allergic disorder treatment.

## 1. Introduction

Allergic diseases are global concerns that roughly account for 25% of healthcare spending in developed countries. Exposure to allergens causes hypersensitivity of the immune system to release immunoglobulin E (IgE), which binds to a high-affinity IgE receptor (FcεRI) on mast cells and basophils [1]. This binding increases intracellular Ca^2+^ level, resulting in cell degranulation, activation of the calcium/nuclear factor of activated T-cells (NFAT) pathway, and, eventually, the release of β-hexosaminidase, histamine, and a variety of inflammatory mediators [2]. Histamine has a key pathological function in many allergic diseases, including atopic dermatitis, allergic rhinitis, and urticaria due to activation of its distinct receptors [3]. Corticosteroids and antagonists are widely used to treat allergic diseases but are not completely effective. Thus, new therapeutic approaches have been developed to fight histamine-mediated allergies through inhibition of the activation of histamine receptors (HRs) and modulation of histidine decarboxylase (HDC) expression through an exclusive enzyme catalyzed for histamine synthesis using natural products.

During an allergic reaction, activation of histamine H1 receptor (H1R) enhances the movement of T helper 2 (Th2) cells is response to the allergen. In addition, H4R-mediated mast cells are activated to modulate powerful inflammatory cascades by discharging inflammatory mediators that stimulate migration of immune cells into the inflammatory sites [4,5]. Commercially available anti-histamine drugs, such as ketotifen fumarate, are H1R antagonists; however, the most common side effects in long-term clinical trials are sedation and weight gain [6]. Recent studies have reported that H4R antagonists alone or in combination with H1R have shown prominent effects in the treatment of inflammatory disorders, such as allergies, asthma, and atopic dermatitis. Indeed, accompanied administration of H1R with H4R antagonists decreased scratching behavior and itch responses in chronic allergic dermatitis [7]. Therefore, targeting both H1R and H4R has been considered as a novel pharmacological modulator of histamine-mediated immune responses.

In addition to HR-based allergic treatment, regulation of histamine synthesis is an emerging strategy for therapeutic application. HDC is a primary and unique enzyme responsible for catalyzing the decarboxylation of the amino acid L-histidine to form histamine [8,9]. Indeed, *HDC* gene-deficient mice show a lack of histamine-synthesizing activity from histidine, indicating the essential role of HDC in the biosynthesis of histamine [9]. The sensitization of mast cells with IgE induces reactive oxygen species (ROS) formation, activating the mitogen-activated protein kinase (MAPK) signaling pathway to stimulate *HDC* mRNA expression by regulating the expression of several transcription factors, such as kruppel-like factor 4 (KLF4)/specific protein 1 (SP1) and GATA binding protein 2 (GATA2)/microphthalmia-associated transcription factor (MITF) [10,11,12]. In addition, HDC expression can be downregulated by preventing excessive ROS production via the nuclear factor erythroid-2-related factor 2 (Nrf2) signaling pathway—a master regulator of redox homeostasis to suppress oxidative stress by targeting anti-oxidant genes such as heme oxygenase 1 (HO-1) and NAD(P)H: quinone oxidoreductase1 (NQO1) [13,14]. Hence, targeting HDC by regulating related signaling pathways and transcription factors is an effective approach for developing an alternative strategy in allergic response treatment. 

*Myrciaria dubia* (camu-camu) belongs to the Myrtaceae family, mostly distributed in the Amazon rainforests of Peru, Brazil, Venezuela, and Colombia [15]. In recent years, camu-camu has become an economically important fruit species in the Amazon region. Due to the rich vitamin C content, camu-camu fruit is consumed globally in beverages, functional foods, and cosmetics in different forms of frozen pulp, juice, and extract [16]. Along with anti-oxidant activities, camu-camu has shown pharmacological effects, including those that are anti-inflammatory, anti-microbial, anti-hypertensive, and anti-obesity [15,16,17,18]. In a previous study, we reported the anti-inflammatory activities of camu-camu fruit extract by modulating MAPK and NFAT signaling pathways in keratinocytes [19]. Despite exerting various pharmacological effects, the anti-allergic effect of camu-camu fruit has not been studied. 

Thus, the present study aimed to evaluate the anti-allergic effect of camu-camu fruit extract by inhibiting mast cell degranulation and targeting novel therapeutic strategies for activation of H1R, H4R, and HDC through the underlying molecular mechanisms of A23187-induced allergies in RBL-2H3 cells. 

## 2. Materials and Methods

### 2.1. Materials

Calcium ionophore (A23187), tacrolimus, and ketotifen fumarate were supplied from Sigma-Aldrich (St. Louis, MO, USA). Primary antibodies were purchased from Cell Signaling (Danvers, MA, USA), Santa Cruz Biotechnology (Santa Cruz, CA, USA), Biovision (Milpitas, CA, USA), and Abcam (Cambridge, UK). 

### 2.2. High-Performance Liquid Chromatography Analysis

The method to identify active components of camu-camu fruit extract was described in a previous study [19]. In brief, HPLC was operated on a Dionex Chromelon TM chromatography data system with P580 and UVD100 detectors (Thermo Fisher Scientific Inc., Waltham, MA, USA). Chromatographic separation was performed on a SUPELCO, Discovery C_18_ column (250 × 4.6 mm, 5 µm particle size); flow rate, 1 mL/min; injection volume, 10 µL, column temperature, 30 °C. An ascorbic acid standard was prepared at concentrations between 1 μg/mL and 500 μg/mL. The chromatographic condition for ascorbic acid was set as follows: 2.5% ethanol in 25 mmol/L sodium dihydrogenphosphate (*v*/*v*); detection wavelength, 256 nm. The cyanidin-3-glycoside standard was diluted in a range of 0.1 to 50 μg/mL. The mobile phase was composed of (A) 0.1% formic acid in water and (B) 0.1% formic acid in acetonitrile. The gradient was linearly increased from 5% B to 95% B over 40 min. The absorbance wavelength of the cyanidin-3-glycoside was detected at 520 nm.

### 2.3. Sample Preparation

Dried camu-camu fruit supplied from Mountain Rose Herbs Co., (Eugene, OR, USA) was prepared in 70% ethanol with 1% formic acid and shaken for 24 h on an SHO-1D digital orbital shaker (Daihan, Korea) at room temperature. The mixture was then filtered and evaporated on a rotary vacuum evaporator at 40 °C (EYELA WORLD—Tokyo Rikakikai Co., Ltd., Tokyo, Japan). The extract yielded was 19.96%.

### 2.4. Cell Culture, Camu-Camu Fruit Treatment, and Stimulation

The basophilic leukemia RBL-2H3 cell line (ATCC CRL-2256) was obtained from American Type Culture Collection (ATCC, VA, USA). Cells were maintained in an incubator with 5% CO_2_ at a temperature of 37 °C on minimum essential medium Eagle (MEM) (Sigma-Aldrich, St. Louis, MO, USA) containing 10% fetal bovine serum plus 1% penicillin/streptomycin (Gibco, Rockville, MD, USA). RBL-2H3 cells were seeded at a density of 3.5 × 10^5^ cells/mL for 24 h. Cells were incubated with supplemented MEM and camu-camu fruit extract (concentrations between 1 and 100 µg/mL) for 1 h before being induced with 100 nM A23187 for 6 h.

### 2.5. Cell Viability Assay

The viability of RBL-2H3 cells was verified by 3-(4,5-dimethylthiazol-2-yl)-2, 5-diphenyltetrazolium bromide (MTT) assay. Briefly, 0.1 mg/mL final concentration of MTT reagent was added to cell culture and incubated for 3 h at 37 °C in dark conditions. After that, supernatants were removed and 100 μL of dimethyl sulfoxide (DMSO) was used to dissolve formazan crystals. The detection wavelength was measured at 570 nm using a FilterMax F5 microplate reader.

### 2.6. β-Hexosaminidase Release Assay

After being seeded for 24 h, cells were washed twice with Siraganian buffer before treatment with samples and A23187. To discontinue the reactions, the plate was placed on ice for 10 min. Next, 50 μL of supernatant was transferred to a new 96-well plate and incubated with 50 μL p-NAG substrate (1 mM p-nitrophenyl-N-acetyl-β-D-glucosaminide in 0.1 M citrate buffer, pH 4.5) for 1 h at 37 °C. Finally, the reaction was terminated by adding 200 μL of stop solution (0.1 M Na_2_CO_3_/NaHCO_3_, pH 10.2) and the absorbance wavelength was measured at 405 nm. The β-hexosaminidase release was determined as the following equation:

$$\beta -\mathrm{hexosaminidase}\;\mathrm{release}\;\mathrm{activity}\;\left(\%\right)=\frac{\mathrm{ODx}}{\mathrm{ODo}}\times 100$$

ODx stands for an optical density of the sample. 

ODo stands for an optical density of the negative control.

### 2.7. Histamine Release Assay

Cells were seeded for 24 h and treated with sample then stimulated with 100 nM A23187. After 6 h of stimulation with A23187, 1 mL of cell culture supernatants was collected and cell pellets were discarded. These supernatants were utilized for histamine assessment. The release of histamine was analyzed with a commercial ELISA kit (Histamine ELISA kit; Abcam, Cambridge, UK) according to the manufacturer’s instruction.

### 2.8. Measurement of Intracellular ROS Production 

After the indicated treatment time, cells were dyed with 30 μM 2′7′-dichlorofluorescein diacetate (DCF-DA) (Sigma-Aldrich) for 30 min at 37 °C in dark conditions. The cells were rinsed twice with cooled 1X PBS and collected using 0.25% trypsin EDTA (Gibco, Rockville, MD, USA). Cells were resuspended in 0.5 mL 1X PBS and measured using flow cytometry (FACSCalibur™; Becton-Dickinson, San Jose, CA, USA). The number of cells is plotted versus the dichlorofluorescein fluorescence detected by the FL-2 channel. The relative ROS production of cells appears in each histogram and is presented in graph form using GraphPad Prism program.

### 2.9. Reverse Transcription-Polymerase Chain Reaction 

TRIzol (Invitrogen Co., Grand Island, NY, USA) was used to extract cellular RNA as in a previous study [20]. Briefly, 2 µg mRNA was quantified and reverse transcribed. PCR amplification was conducted using PCR premix (Bioneer, Korea) and a Veriti Thermal Cycler (Applied Biosystems, Foster City, CA, USA). The primers used for *H1R*, *H4R*, and *HDC* are indicated in Supplementary Material Table S1. The PCR products were separated using agarose gel electrophoresis and visualized under UV illumination.

### 2.10. Western Blot Analysis

Cells were suspended overnight in RIPA lysis buffer obtained from Sigma-Aldrich. After centrifugation at 12,000 rpm for 15 min, cell lysates were collected and calibrated in an equivalent amount of protein using a Bradford reagent (Bio-Rad, Hercules, CA, USA) and bovine serum albumin (BSA) as the standard. The same quantities of protein were loaded into sodium dodecyl sulfate polyacrylamide gel electrophoresis (SDS-PAGE) gel for separation and transferred to a PVDF membrane (Bio-Rad, Hercules, CA, USA). The membrane was blockaded with 5% BSA or 5% skim milk prepared in 1X TBST. The primary antibody was supplemented to the membrane and shaken overnight at 4 °C. The membrane was immersed in secondary antibody solution, and the level of protein production was specified using electrochemiluminescence (ECL) detection reagents (Fujifilm, LAS-4000, Tokyo, Japan).

### 2.11. Statistical Analysis

The data represent three individual experiments and are presented as mean ± standard deviation (SD). Statistical comparison between treatments was performed using one-way ANOVA followed by Duncan’s test. For statistical analysis, Student’s *t* test was applied to compare individual treatments to the controls. The level of statistical significance was set as follows: ^#^ *p* < 0.05, ^##^ *p* < 0.01, and ^###^ *p* < 0.001 vs. the control group, * *p* < 0.05, ** *p* < 0.01, and *** *p* < 0.001 vs. the A23187-treated group.

## 3. Results

### 3.1. Quantification of Active Components from Camu-Camu Fruit Extract

**Figure 1:**
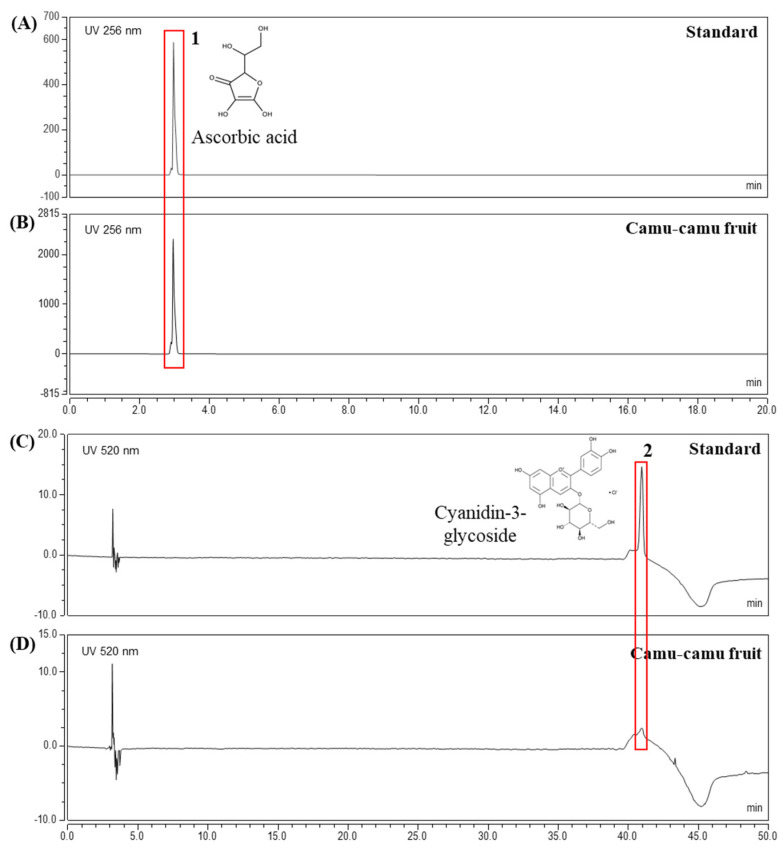
HPLC analysis of ascorbic acid standard (**A**), cyanidin-3-glycoside standard (**C**), and camu-camu fruit extract (**B**,**D**).

The extract yielded was 19.96%. HPLC analysis was performed to determine the contents of active components in camu-camu fruit extract. The chromatogram in Figure 1 confirmed ascorbic acid (3.1 min) and cyanidin-3-glycoside (40.96 min) as active compounds at concentrations of 47.7 mg/g and 0.1 mg/g, respectively. In addition, Supplementary Material Figure S1 presents the HPLC results of ellagic acid and quercetin in the same extract of camu-camu fruit [19]. This elucidated the presence of various active components in camu-camu fruit extract.

### 3.2. Effects of Camu-Camu Fruit Extract on Cell Viability, β-Hexosaminidase, and Histamine Release

**Figure 2:**
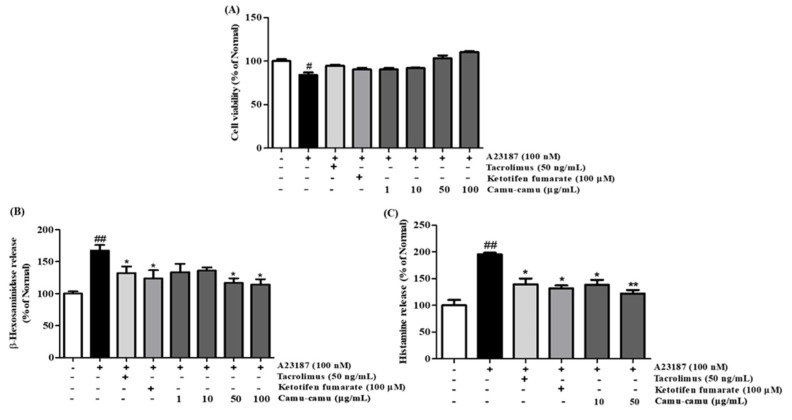
Effect of camu-camu fruit extract on cell viability (**A**), β-hexosaminidase (**B**), and histamine release (**C**) in A23187-induced RBL-2H3 cells. Tacrolimus and ketotifen fumarate were employed as positive controls. All data are displayed as mean ± standard deviation (SD) of three independent experiments (^#^ *p* < 0.05 and ^##^ *p* < 0.01 vs. the control group, * *p* < 0.05 and ** *p* < 0.01 vs. the A23187-treated group).

To verify the effect of camu-camu fruit extract on cell viability, an MTT assay was performed. As presented in Supplementary Material Figure S2, at a concentration of 50 µg/mL, cell viability of camu-camu fruit extract remained at 90.2%, whereas 100 µg/mL of extract reduced cell viability to 42.8% in RBL-2H3 cells. In Figure 2A, A23187-induced RBL-2H3 cells showed a noticeable decrease in cell viability compared with non-treated cells, whereas pretreatment with camu-camu fruit extract ameliorated this decrease in a dose-dependent manner. There were no significant changes in cell viability when cells were exposed to positive controls of tacrolimus and ketotifen fumarate. This indicates that camu-camu fruit extract is safe for cells and 50 µg/mL camu-camu fruit extract was used as the highest concentration for further experiments.

To confirm whether camu-camu fruit extract suppressed cell degranulation, the β-hexosaminidase assay was conducted in A23187-induced RBL-2H3 cells. Application of tacrolimus and ketotifen fumarate decreased the release of β-hexosaminidase. Pretreatment with camu-camu fruit extract markedly inhibited the release of β-hexosaminidase and showed better inhibition than that of the positive control (Figure 2B). 

Histamine release in A23187-stimulated cells increased 95.5% compared with that of non-treated cells (Figure 2C). Treatment of camu-camu fruit extract inhibited this release in a dose-dependent manner. These data indicate that camu-camu fruit extract decreased cell degranulation and histamine release.

### 3.3. Effect of Camu-Camu Fruit Extract on Intracellular ROS Production 

**Figure 3:**
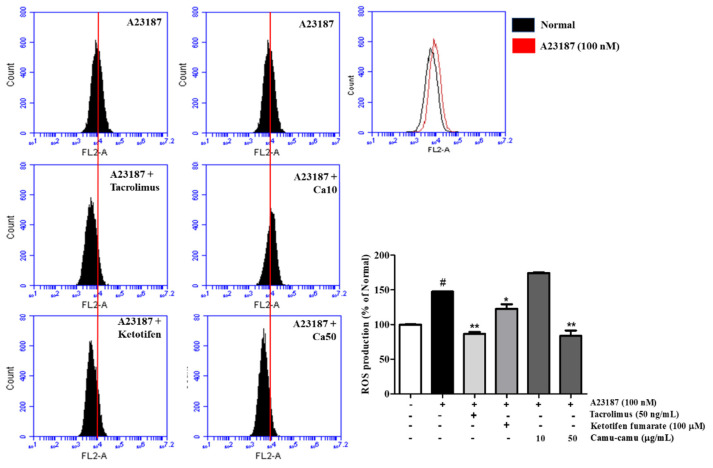
Effect of camu-camu fruit extract on intracellular ROS production in A23187-induced RBL-2H3 cells. Levels of intracellular ROS production were measured by flow cytometry. All data are displayed as mean ± SD of three independent experiments (^#^ *p* < 0.05 vs. the control group, * *p* < 0.05 and ** *p* < 0.01 vs. the A23187-treated group).

As shown in Figure 3, sanitization of A23187 stimulated ROS overproduction to 147.3% compared with that of non-induced cells. To confirm the effect of camu-camu fruit extract on reducing intracellular ROS generation, 10 and 50 µg/mL samples of camu-camu fruit extract were assessed either with or without A23187 sanitization. In Supplementary Material Figure S3, intracellular ROS generation decreased in dose-dependent manner. Indeed, 10 and 50 µg/mL samples of camu-camu fruit extract caused a reduction of 33.8% and 41.6%, respectively, compared with non-treated cells. As shown in Figure 3, pretreatment with 50 µg/mL camu-camu fruit extract reduced ROS overproduction by 43.2% compared to that in A23187-treated cells without pretreatment and showed better inhibition than that of the positive controls. In particular, camu-camu fruit extract treatment decreased ROS production by 31.8% compared to that of ketotifen fumarate-treated cells. This suggests that camu-camu fruit extract suppresses oxidative stress better than positive controls in A23187-induced RBL-2H3 cells.

### 3.4. Effect of Camu-Camu Fruit Extract on mRNA Expression of H1R and H4R

**Figure 4:**
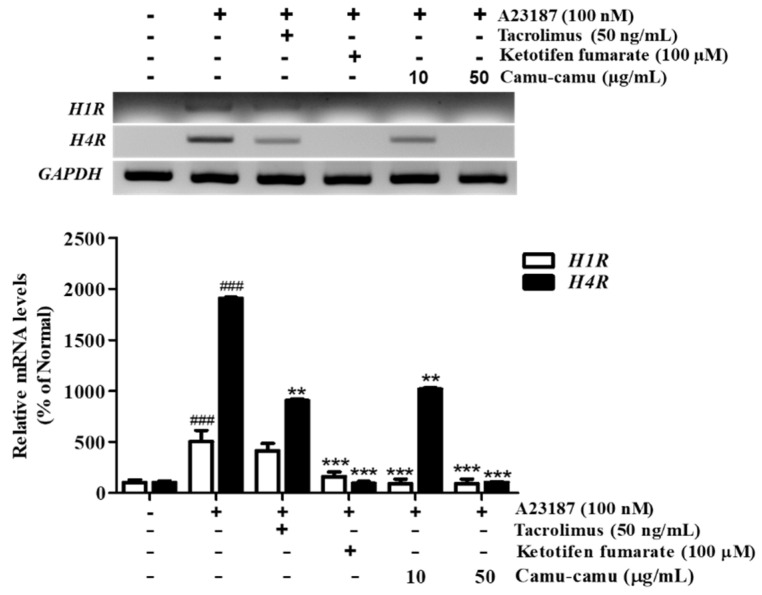
Effect of camu-camu fruit extract on mRNA expression of *H1R* and *H4R* in A23187-induced RBL-2H3 cells. Normalized mRNA amount was relatively compared to *GAPDH*. The results were calculated as percentage of non-treated cells and displayed as mean ± SD of three independent experiments (^###^ *p* < 0.001 vs. the control group, ** *p* < 0.01 and *** *p* < 0.001 vs. the A23187-treated group).

Even though the role of H4R is not fully understood, it has been shown as a potential target for allergic inflammation treatment. We verified inhibition of mRNA expression of H4R and H1R by camu-camu fruit extract. Figure 4 shows that mRNA expression of *H4R* was stronger than that of *H1R* in RBL-2H3 cells. While the expression of *H1R* increased to 503.2% in A23187-treated cells, the expression of *H4R* increased to 1904.2% compared to that of non-treated cells. However, camu-camu fruit extract markedly inhibited the expression of both *H1R* and *H4R*. Treatment with camu-camu fruit extract at 50 µg/mL decreased the mRNA levels of *H1R* and *H4R* by 82.6% and 94.6%, respectively, compared with that of A23187-treated cells. Ketotifen fumarate acts as a second-generation non-competitive H1 anti-histamine and showed strong inhibitory effects on the expression of H1R. The results indicate that H4R is expressed strongly on mast cells, and that camu-camu fruit extract inhibited mRNA expression of both *H1R* and *H4R*.

### 3.5. Effect of Camu-Camu Fruit Extract on mRNA and Protein Expression of HDC

**Figure 5:**
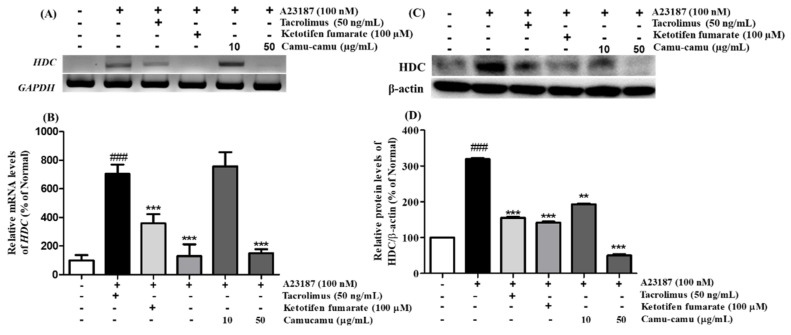
Effect of camu-camu fruit extract on mRNA (**A**,**B**) and protein expression (**C**,**D**) of HDC in A23187-induced RBL-2H3 cells. mRNA level was normalized to *GAPDH*, while protein level was normalized to β-actin. The results were calculated as the percentage of non-treated cells and displayed as mean ± SD of three independent experiments (^###^ *p* < 0.001 vs. the control group, ** *p* < 0.01 and *** *p* < 0.001 vs. the A23187-treated group).

Histidine decarboxylase (HDC) is a primary enzyme responsible for catalyzing histidine decarboxylase to histamine to perform its versatile functions in regulating allergic responses. Herein, we confirmed the mRNA and protein expression of HDC on A23187-induced allergies in RBL-2H3 cells. A23187 treatment induced significant mRNA and protein expression of HDC. Under exposure to A23187, mRNA and protein expression of HDC increased to 705.7% and 219.4%, respectively, compared to non-treated cells. This increase was blocked by both positive controls and 50 µg/mL camu-camu fruit extract treatment. While tacrolimus and ketotifen fumarate showed almost the same level of inhibition of HDC activity, 50 µg/mL camu-camu fruit extract showed a much greater effect. In particular, 50 µg/mL camu-camu fruit extract suppressed 83.2% and 84.2% of accumulated HDC mRNA and protein expression, respectively, compared with that of A23187-treated cells (Figure 5). 

### 3.6. Effect of Camu-Camu Fruit Extract on Calcium Channel Protein Expression

**Figure 6:**
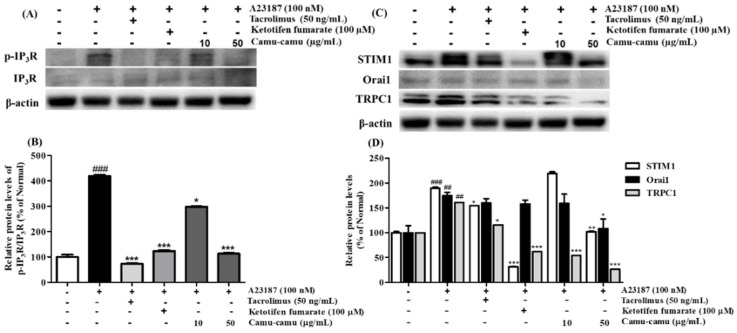
Effect of camu-camu fruit extract on IP_3_R phosphorylation (**A**,**B**) and calcium channel proteins (**C**,**D**) in A23187-induced RBL-2H3 cells. Band intensities were quantified by densitometry, normalized to the level of β-actin. Then, it was calculated as the percentage of the untreated cells and displayed as mean ± SD of three independent experiments (^##^ *p* < 0.01 and (^###^ *p* < 0.001 vs. the control group, * *p* < 0.05, ** *p* < 0.001 and *** *p* < 0.001 vs. the A23187-treated group).

In mast cells, mobilization of intracellular Ca^2+^ level regulates cell degranulation through activation of store-operated Ca^2+^ entry (SOCE) channels. To investigate the effect of camu-camu fruit extract on the expression of calcium-related protein expression, RBL-2H3 cells were stimulated with 100 nM A23187. As shown in Figure 6, the levels of phosphorylated IP_3_R, STIM1, Orai1, and TRPC1 were upregulated in cells stimulated with A23187. However, 50 μg/mL camu-camu fruit extract inhibited the levels of p-IP_3_R, STIM1, Orai1, and TRPC1 by 73.1%, 46.1%, 38.1%, and 83.7%, respectively, compared to that of A23187-treated cells. This trend was also seen in the way that camu-camu fruit extract inhibited STIM1 expression in dose-dependent manner in RBL-2H3 cells, as shown in Supplementary Material Figure S4. The positive controls also inhibited the phosphorylation of IP_3_R and calcium channel proteins. These results indicate that camu-camu fruit extract blocks the extracellular calcium influx via downregulation of calcium channel proteins to reduce the release of histamine.

### 3.7. Effect of Camu-Camu Fruit Extract on MAPK Activation

**Figure 7:**
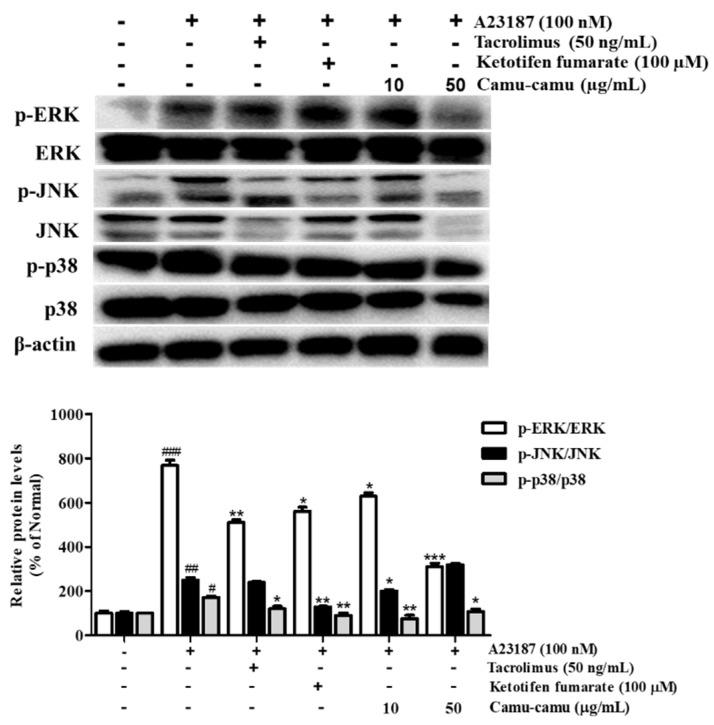
Effect of camu-camu fruit extract on MAPK activation in A23187-induced RBL-2H3 cells. Band intensities were quantified by densitometry, normalized to the level of β-actin. Then, it was calculated as a percentage of non-treated cells and displayed as mean ± SD of three independent experiments (^#^ *p* < 0.05, ^##^ *p* < 0.01, and ^###^ *p* < 0.001 vs. the control group, * *p* < 0.05, ** *p* < 0.001 and *** *p* < 0.001 vs. the A23187-treated group).

To determine the mechanism responsible for the inhibitory effect of camu-camu fruit extract on histamine receptors and HDC, the phosphorylation of MAPK was investigated. Different extract concentrations of camu-camu fruit extract were tested on non-treated and A23187-treated cells to see its influence on phosphorylation of ERK, JNK, and p38. As shown in Supplementary Material Figure S5, the expression of p-ERK and p-JNK was impaired by the treatment of camu-camu fruit extract in concentrations of both 10 and 50 µg/mL, while the expression of p-p38 did not show any difference. This indicates the potential protection of camu-camu fruit extract in RBL-2H3 cells. With exposure to A23187, the phosphorylation of ERK, JNK, and p38 increased to 767.8%, 250.5%, and 172% compared to that of non-treated cells, respectively. However, these increases were reversed by treatment with tacrolimus and ketotifen fumarate positive controls. Camu-camu fruit extract treatment showed the same effect as positive controls, with 50 µg/mL camu-camu fruit decreasing p-ERK and p-p38 by 59.5% and 37.5%, respectively, compared with that of A23187-treated cells (Figure 7). These results suggest that camu-camu fruit extract inhibited the activation of H1R, H4R, and HDC expression by targeting MAPK. 

### 3.8. Effect of Camu-Camu Fruit Extract on the Expression of KLF4/SP1 and GATA2/MITF Transcription Factors

**Figure 8:**
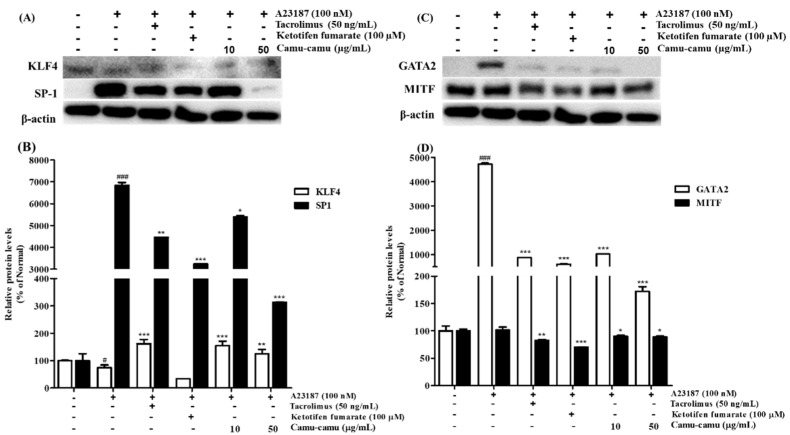
Effect of camu-camu fruit extract on KLF4/SP1 (**A**,**B**) and GATA2/MITF (**C**,**D**) expression in A23187-induced RBL-2H3 cells. Band intensities of KLF4, SP1, GATA2, and MITF were quantified by densitometry, normalized to the level of β-actin. Then, it was calculated as a percentage of non-treated cells and displayed as mean ± SD of three independent experiments (^#^ *p* < 0.05 and ^###^ *p* < 0.001 vs. the control group, * *p* < 0.05, ** *p* < 0.001 and *** *p* < 0.001 vs. the A23187-treated group).

MAPK was reported to stimulate HDC mRNA expression and consequent histamine production by regulating the expression of several transcription factors. Thus, the effect of camu-camu fruit extract on HDC expression was investigated using transcription factors KLF4, SP1, GATA2, and MITF [12,21]. Under stimulation with A23187, the expression of KLF4 was impaired by 25.1%, while the level of SP1 was overexpressed by 6738.5% compared to that of non-stimulated cells. However, it was reversed by treatment with camu-camu fruit extract. Camu-camu fruit extract upregulated the level of KLF4 up to 206.7% and suppressed the expression of SP1 by 95.4% at a concentration of 50 μg/mL compared to that of A23187-stimulated cells. On the contrary, ketotifen fumarate was only capable of suppressing SP1 expression (Figure 8A,B). 

GATA2 was demonstrated to regulate the transcription factor MITF. Thus, the effects of camu-camu fruit extract on regulating HDC expression were also investigated on GATA2 and MITF transcription factors. Compared to non-treated cells, A23187-treated cells stimulated GATA2 expression to 4723.3% but MITF expression did not show much difference. Treatment with tacrolimus and ketotifen fumarate successfully inhibited the level of GATA2 and MITF expression. Compared to positive controls, camu-camu fruit extract treatment showed a greater effect on inhibiting GATA2 overexpression. For instance, camu-camu fruit extract dose-dependently impaired GATA2 expression by 78.3% and 96.4% at the concentration of 10 and 50 µg/mL, respectively (Figure 8C,D).

### 3.9. Effect of Camu-Camu Fruit Extract on Nrf2 Activation

**Figure 9:**
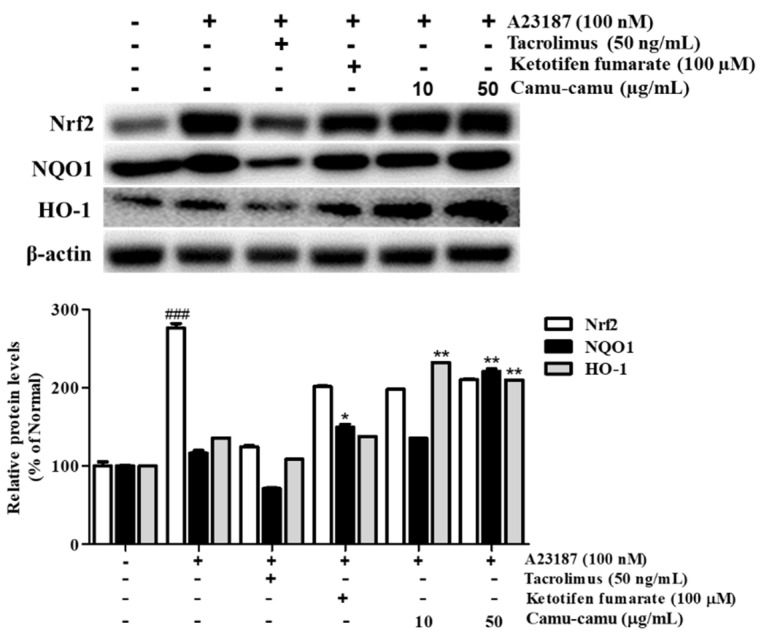
Effect of camu-camu fruit extract on Nrf2 activation in A23187-induced RBL-2H3 cells. Band intensities were quantified by densitometry, normalized to the level of β-actin. Then, it was calculated as a percentage of non-treated cells and displayed as mean ± SD of three independent experiments (^###^ *p* < 0.001 vs. the control group, * *p* < 0.05 and ** *p* < 0.001 vs. the A23187-treated group).

Under oxidative stress, the transcription factor Nrf2 is activated to upregulate the expression of anti-oxidant genes, including HO-1 and NQO1. This upregulation protects the cells against oxidative stress [22]. Sanitization of A23187 increased the expression of Nrf2, HO-1, and NQO1 to 276.2%, 136.2%, and 116.5%, respectively, compared with that of non-treated cells. To evaluate whether camu-camu fruit extract balances oxidative stress, the expression of Nrf2, HO-1, and NQO1 was investigated in A23187-stimulated RBL-2H3 cells. As shown in Figure 9, 50 µg/mL camu-camu fruit extract promoted the expression of NQO1 and HO-1 by 160.1% and 203.2% compared with that of A23187-treated cells, respectively, while the expression of Nrf2 did not show any significant increase.

### 3.10. Effect of Camu-Camu Fruit Extract on NFAT Activation

**Figure 10:**
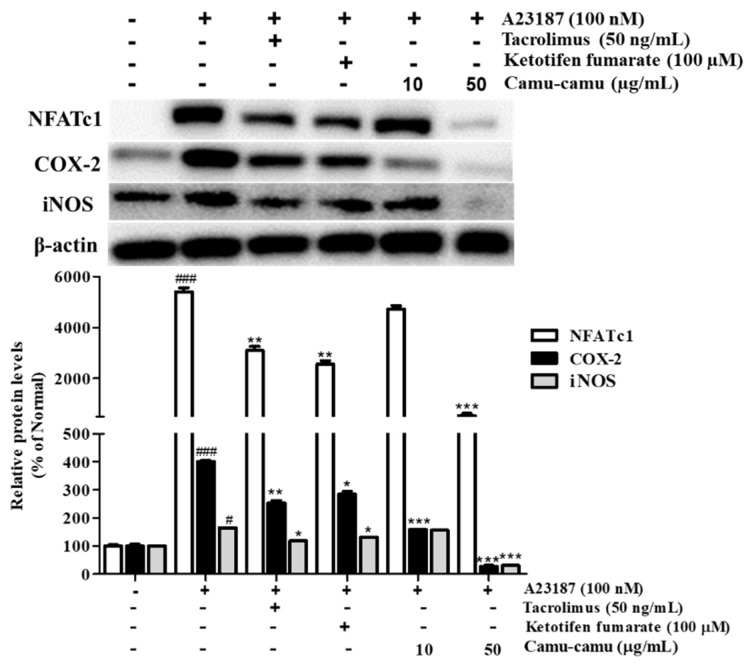
Effect of camu-camu fruit extract on NFAT activation in A23187-induced RBL-2H3 cells. Band intensities were quantified by densitometry, normalized to the level of β-actin. Then, it was calculated as a percentage of non-treated cells and displayed as mean ± SD of three independent experiments (^#^ *p* < 0.05 and ^###^ *p* < 0.001 vs. the control group, * *p* < 0.05, ** *p* < 0.001 and *** *p* < 0.001 vs. the A23187-treated group).

NFAT is a calcium/calcineurin-dependent transcription factor that plays an important role in cytokine transcription regulation in mast cells. To investigate NFATc1 expression on A23187-stimulated RBL-2H3 cells, cells were treated with 100 nM A23187 for 6 h. The level of NFATc1 increased by 5302.4%. As a calcineurin inhibitor, tacrolimus showed better suppression of NFATc1, COX-2, and iNOS compared to ketotifen fumarate. Camu-camu fruit extract significantly inhibited the expression of NFATc1, COX-2, and iNOS. In particular, pre-incubation with 50 µg/mL camu-camu fruit extract decreased the expression of NFATc1, COX-2, and iNOS by 90%, 93.3%, and 81.1% compared with that of A23187-treated cells, respectively (Figure 10). 

## 4. Discussion

Camu-camu fruit extract contains various active compounds, especially vitamin C and cyanidin-3-glycoside, which contribute to the anti-oxidant and anti-inflammatory activities. The present study demonstrated the potential anti-allergic effect of camu-camu fruit extract in A23187-induced RBL-2H3 cells by inhibiting mast cell degranulation and targeting HRs and HDC expression, presenting novel therapeutic strategies on allergic treatment. Therefore, camu-camu fruit extract is a promising candidate for an alternative drug.

**Figure 11:**
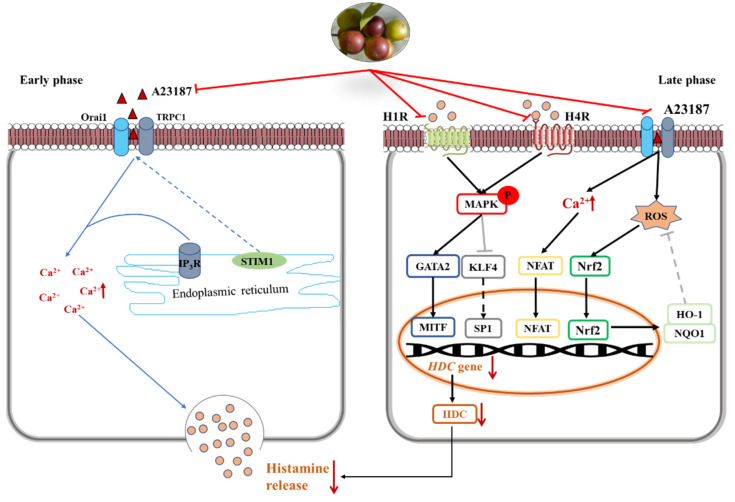
Inhibitory effect of camu-camu fruit extract on mast cells via regulation of H1R and H4R.

In the early phase, stored histamine is released via cell degranulation within minutes [23]. Mast cell activation results in a decrease of intracellular Ca^2+^ level. The depletion of intracellular Ca^2+^ level leads to the entry of extracellular Ca^2+^ and subsequently causes the fusion of preformed granules and plasma membrane [2]. In the present study, camu-camu fruit extract played important roles in immediate allergic reactions and calcium-dependent mast cell degranulation. It was verified that camu-camu fruit extract significantly reduced the phosphorylation of IP_3_R and calcium-dependent proteins such as STIM1, Orai1, and TRPC1. Moreover, the increase of the calcium channel also promotes translocation of NFAT to the nucleus from the cytoplasm [24]. Calcineurin/NFAT are important for the production of various cytokines, such as IL-4 and IL-13, which are essential for productive immune defense [25]. However, camu-camu fruit extract dose-dependently inhibited the expression of NFATc1, COX-2, and iNOS. In order to validate the inhibitory effect of mast cell degranulation, camu-camu fruit extract was compared to tacrolimus, a calcineurin inhibitor widely used to treat T-cell-mediated diseases. The results showed that camu-camu fruit extract was more effective both in suppressing calcium-dependent proteins and in activating NFAT signaling. These results suggest that camu-camu fruit extract suppresses exocytosis of mast cell granules by regulating the calcium/NFAT signaling pathway to reduce the release of histamine and β-hexosaminidase (Figure 11).

Histamine receptors, especially H1R and H4R, have a critical role in pathological processes of allergic inflammatory responses. Thus, for treatment of inflammatory disorders, H4R has been considered a novel pharmacological modulator of histamine-mediated immune responses. In the present study, the expression of H4R was more than three times higher than that of H1R; however, camu-camu fruit extract successfully inhibited mRNA expression of both *H1R* and *H4R*. Ketotifen fumarate is a second-generation non-competitive H1 anti-histamine and mast cell stabilizer that is frequently used in the treatment of allergic conjunctivitis, asthma, atopic dermatitis, and allergic rhinitis. Even though ketotifen fumarate is an H1R antagonist, camu-camu fruit extract showed a superior effect in inhibiting the expression of H1R. By targeting both H1R and H4R, camu-camu fruit extract blocked the phosphorylation of MAPK. On the other hand, mast cell activation regulates calcium influx, which interacts and contributes to ROS production. Thus, an increased level of Ca^2+^ induces oxidative stress and reduces anti-oxidant capacity [26,27]. ROS represents oxidative stress and plays an essential role on different cellular processes, both physiological, such as neuromodulation, and pathological, such as ischemia and various neurodegenerative disorders, alongside its role in moderating hormesis [28,29]. However, HO-1 and NQO1 are cytoprotective enzymes against oxidative stress and are regulated by the transcription factor Nrf2. Nrf2 mediates endogenous cellular defense pathways that integrate adaptive stress responses in the prevention of an imbalance of oxidative stress and neurodegenerative diseases [29,30]. In this study, camu-camu fruit extract reduced excessive intracellular ROS production caused by an increase of HO-1 and NQO1. Furthermore, as camu-camu fruit extract has been reported to inhibit high glucose-induced inflammation through the regulation of MAPK and Nrf2, it was verified that camu-camu fruit extract inhibited HR-mediated mast cell activation by suppressing MAPK and elevating Nrf2 signaling pathways (Figure 11).

In the late phase, histamine is continuously synthesized via HDC induction together with increased secretion of cytokines characterized by Th2 response. Thus, agents that inhibit HDC synthesis have drawn great attention as an effective strategy for treatment of allergic disorders. A previous study reported that the expression of *HDC* mRNA and protein was inhibited by the MAPK inhibitor, indicating that MAPK plays an essential role in the synthesis of HDC [31]. By targeting MAPK, camu-camu fruit extract suppressed the mRNA expression of *HDC* and decreased HDC enzyme production. MAPK mediated the expression of HDC by regulating its transcription factors, KLF4, SP1, GATA2, and MITF. Nishimura et al. (2020) reported that KLF4 is required for suppression of *HDC* expression in bone-marrow-derived mast cells by competing with SP1 in the KLF4/SP1 composite binding site at the promoter GC box [11,21]. GATA2 induces the expression of MITF that is required for anaphylaxis [12]. Due to inhibition of phosphorylated ERK, JNK, and p38, subunits of the MAPK family, camu-camu fruit extract significantly reversed the action of transcription factors and resulted in a decrease of HDC expression. This suggests that camu-camu fruit extract can act as an HDC inhibitor to regulate the release of histamine (Figure 11). 

In order to treat allergic disorders, HR antagonists and mast cell stabilizers (such as ketotifen fumarate) and calcineurin inhibitors (such as tacrolimus) are used; however, long-term use of these drugs causes systemic side effects, including headache, drowsiness, burning sensation, and increased cancer risk [32]. Thus, there is need for a natural product that serves as an anti-allergic therapy with proven efficacy and safety. There have been a number of studies performed on natural products to confirm their anti-allergic effects caused by activation of the H1 receptor [31,32,33]. Nevertheless, most of them were not capable of inhibiting the H1 receptor, with the exception of *Urtica dioica* extract. In another study, Annika et al. (2017) pointed out that crude extracts and defined compounds of *C. longa* have been recognized as potential and reasonable ligand lead structures at the H4 receptor [29]. On the other hand, high-dose vitamin C was reported to be beneficial in patients with allergic diseases due to the protection of blood plasma against oxidative stress [34]. Cyanidin-3-glycoside was indicated to have potential as an anti-allergic agent suppressing Th2 activation [35]. These active components of camu-camu fruit extract suggest potential effects of camu-camu fruit extract in anti-allergic treatment. In the present study, camu-camu fruit extract functioned as a mast cell stabilizer and HR blocker by inhibiting mast cell degranulation and H1R- and H4R-mediated mast cell activation. Moreover, camu-camu fruit extract inhibited HDC expression, resulting in histamine reduction. Taken together, these findings suggest that camu-camu fruit extract can be developed as an alternative candidate to treat allergic disorders.

## 5. Conclusions

The present study was conducted to evaluate the inhibitory effect of camu-camu fruit extract on A23187-induced allergies in RBL-2H3 cells. Our data indicate that camu-camu fruit extract decreased mast cell degranulation by mediating the calcium/NFAT signaling pathway to alleviate allergic response. Camu-camu fruit extract downregulated the MAPK signaling pathway to inhibit H1R and H4R activation, a novel therapeutic approach to target histamine-mediated allergic diseases. By downregulating MAPK phosphorylation, camu-camu fruit extract modulated its transcription factors, such as KLF4/SP1 and GATA2/MITF, to inhibit the expression of HDC and further led to the decrease of histamine synthesis. Camu-camu fruit extract also acted as a strong anti-oxidant, triggering the Nrf2 signaling and cytoprotective enzymes to protect against ROS overproduction in mast cells. 

In conclusion, this work “opens the door” to new investigations using natural sources to isolate bioactive molecules for the treatment of allergies and associated pathologies. The multi-target functions of camu-camu fruit extract suggest that it can be a substitute for commercial synthetic drugs to contribute as an effective candidate in allergic disorder treatment. Nevertheless, in vivo and clinical trials need to be carried out to confirm the effect of camu-camu fruit extract and its bioactive molecules. 

## Data Availability

The data are contained within the article and supplementary materials.

## Supplementary Materials

The following are available online at https://www.mdpi.com/article/10.3390/antiox11010104/s1.
