# Supplementary material for: Anti-Allergic Effects of Myrciaria dubia (Camu-Camu) Fruit Extract by Inhibiting Histamine H1 and H4 Receptors and Histidine Decarboxylase in RBL-2H3 Cells
Source: Antioxidants (Basel). 2021 Dec 31;11(1):104. doi: 10.3390/antiox11010104 (PMC8773304; doi:10.3390/antiox11010104)
Supplement: Supplementary file 1 [file antioxidants-11-00104-s001.zip › Supplementary Materials.pdf]

**Supplementary Material Table S1.** Polymerase chain reaction (PCR) primers used in this experiment

| Cell line | Gene name | Primer | Sequence (5' – 3') | Length (bp) |
|---|---|---|---|---|
| Rat (RBL-2H3) | *H1R* | Forward | TCTGGCTTCCTACCCGTCCT | 328 |
| | | Reverse | TGTCCTGTTCCCCTCACACA | |
| | *H4R* | Forward | ACATCCCTCACACGCTGTTT | 320 |
| | | Reverse | GGCGAGGATGTACCACTCAG | |
| | *HDC* | Forward | TGACAACTTCTCACTCCGAGG | 780 |
| | | Reverse | ACAAGGTTAGCAGCCTCTCG | |
| | *GAPDH* | Forward | TGATGACATCAAGAAGGTGGTGAAG | 240 |
| | | Reverse | TCCTTGGAGGCCATGTAGGCCAT | |

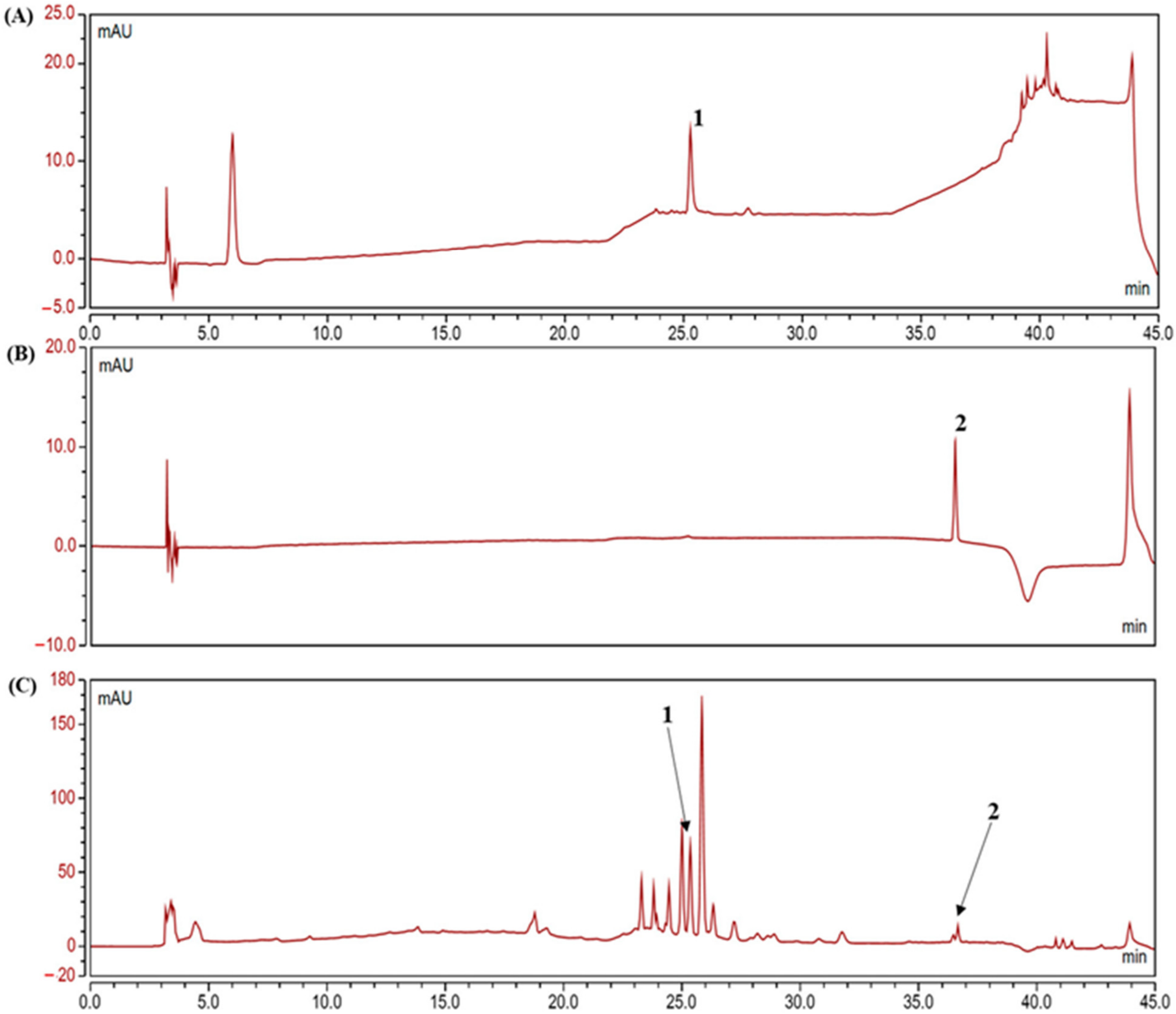


**Supplementary Material Figure S1.** The HPLC (high-performance liquid chromatography) results of ellagic acid (peak 1) and quercetin standards (peak 2) (A, B) and the contents of ellagic acid and quercetin in the camu-camu fruit extract (C). (Do *et al*., 2021).

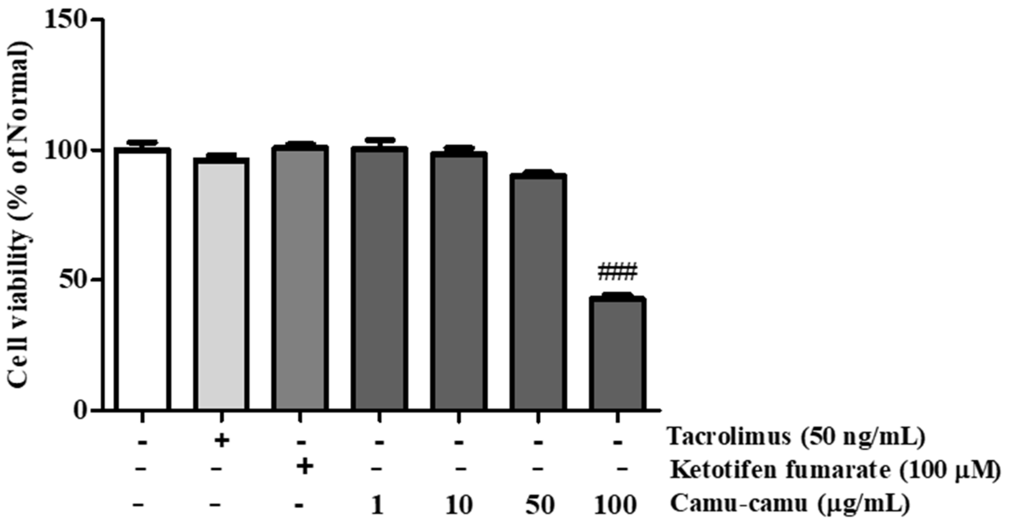


**Supplementary Material Figure S2.** Effect of camu-camu fruit extract on cell viability in RBL-2H3 cells. All data were displayed as mean ± SD of three independent experiments ($^{###}p < 0.001$ vs. the control group).

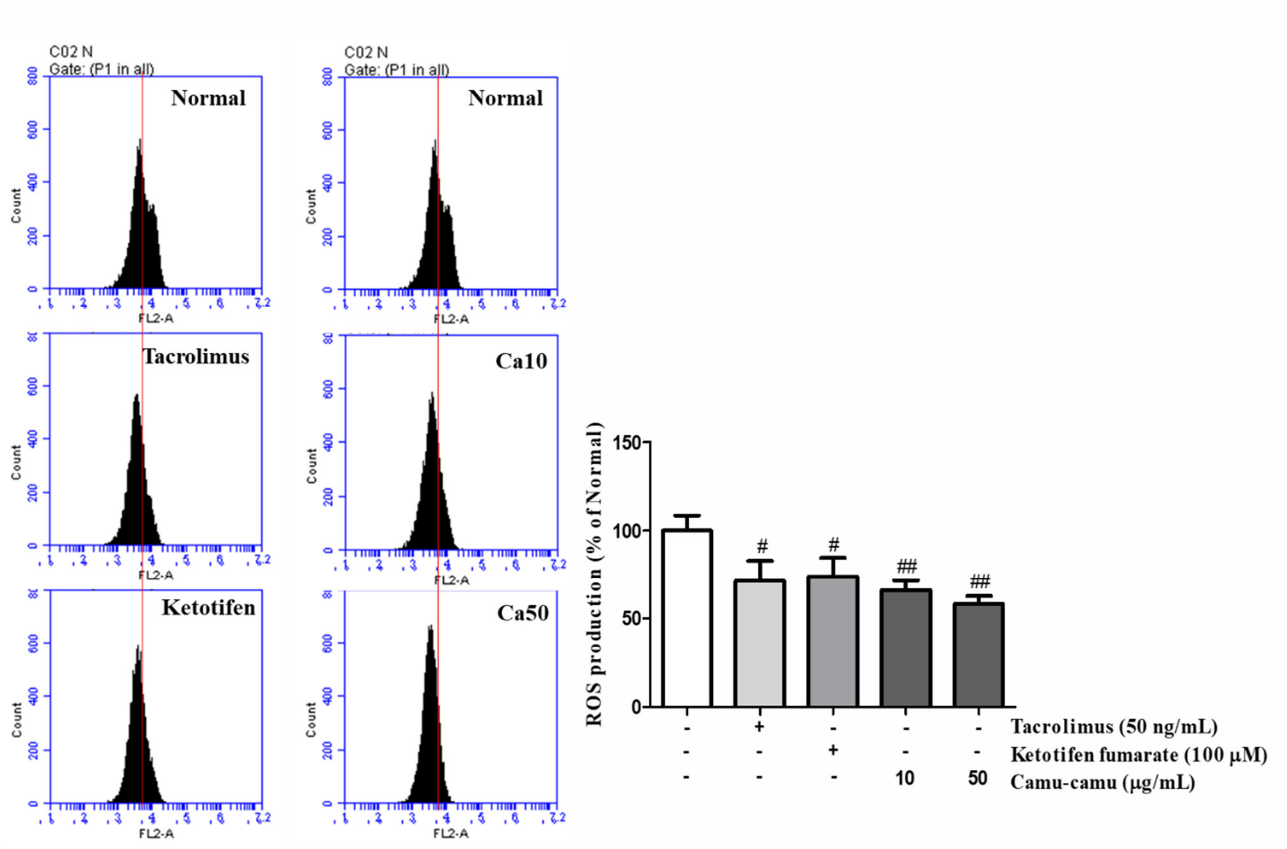


**Supplementary Material Figure S3.** Effect of camu-camu fruit extract on intracellular ROS production in RBL-2H3 cells. Levels of intracellular ROS production were measured by flow cytometry. All data were displayed as mean ± SD of three independent experiments ($^{#}p < 0.05$ and $^{##}p < 0.01$ vs. the control group).

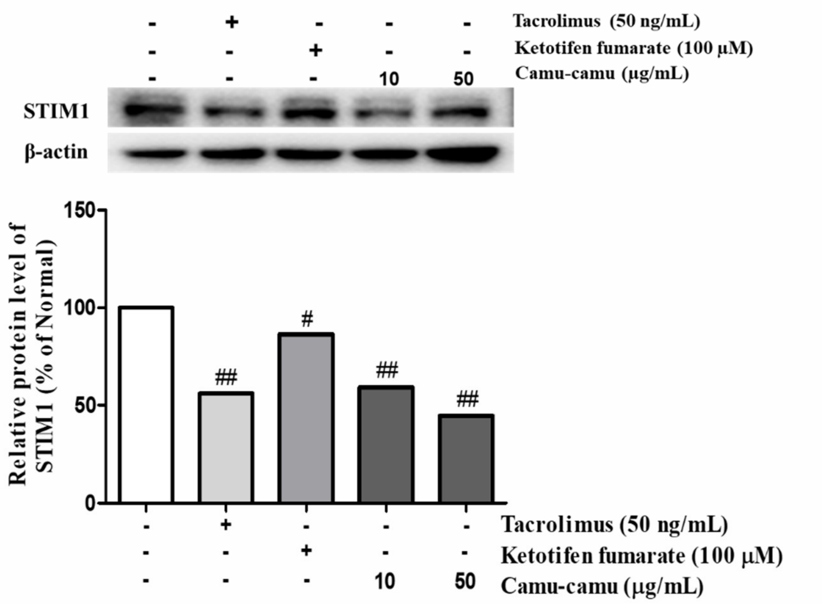


**Supplementary Material Figure S4.** Effect of camu-camu fruit extract on STIM1 expression in RBL-2H3 cells**.** Band intensities were quantified by densitometry, normalized to the level of β-actin. Then it was calculated as the percentage of the untreated cells and displayed as mean ± SD ($^{\#}p < 0.05$ and $^{\#\#}p < 0.01$ vs. the control group).

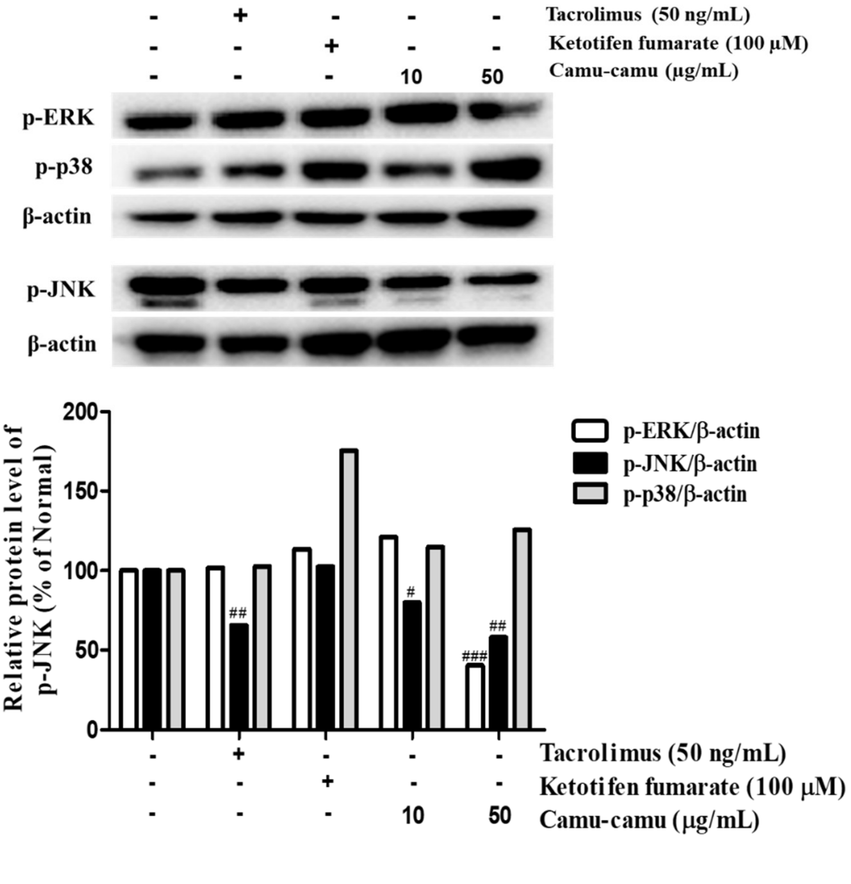


**Supplementary Material Figure S5.** Effect of camu-camu fruit extract on MAPK activation in RBL-2H3 cells. Band intensities were quantified by densitometry, normalized to the level of β-actin. Then it was calculated as a percentage of non-treated cells and displayed as mean ± SD ($^{\#}p < 0.05$, $^{\#\#}p < 0.05$, and $^{\#\#\#}p < 0.001$ vs. the control group).
